# Supplementary material for: No fruits without color: Cross-modal priming and EEG reveal different roles for different features across semantic categories
Source: PLoS One. 2021 Apr 14;16(4):e0234219. doi: 10.1371/journal.pone.0234219 (PMC8046255; doi:10.1371/journal.pone.0234219)
Supplement: S1 Appendix — (DOCX) [file pone.0234219.s001.docx]

**S1 Appendix. Supplementary Data**

**
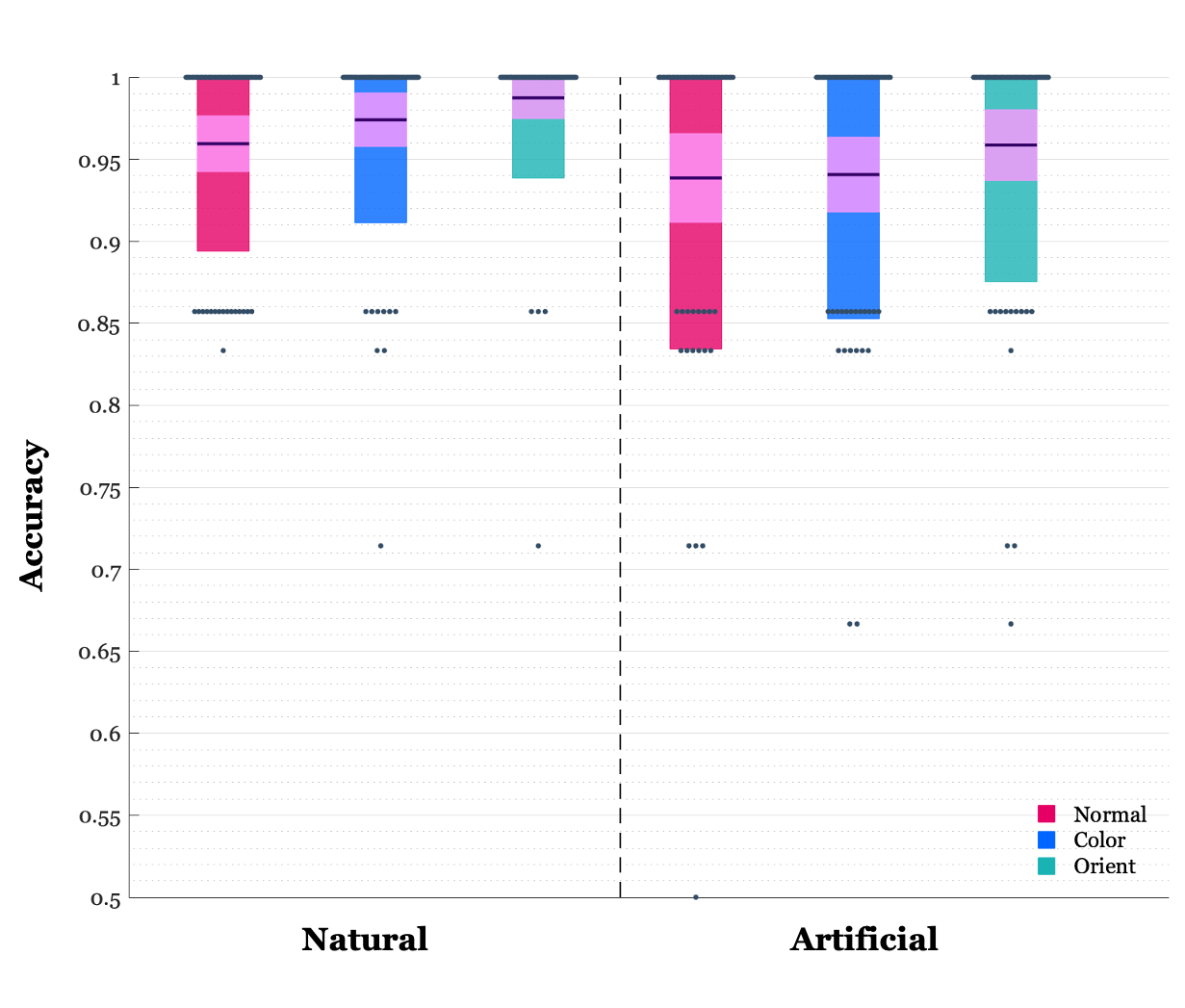
**

Fig. A.1. *Scatterplot of accuracies averaged across all trials per subject, plotted by category and condition.* Each dot represents the mean accuracy for an individual subject. The color of each box corresponds to condition (see legend), the expanse of the box represents one standard deviation, the pink middle strip represents the standard error of the mean (SEM) for the 95% confidence interval, and the purple line represents the mean.

**
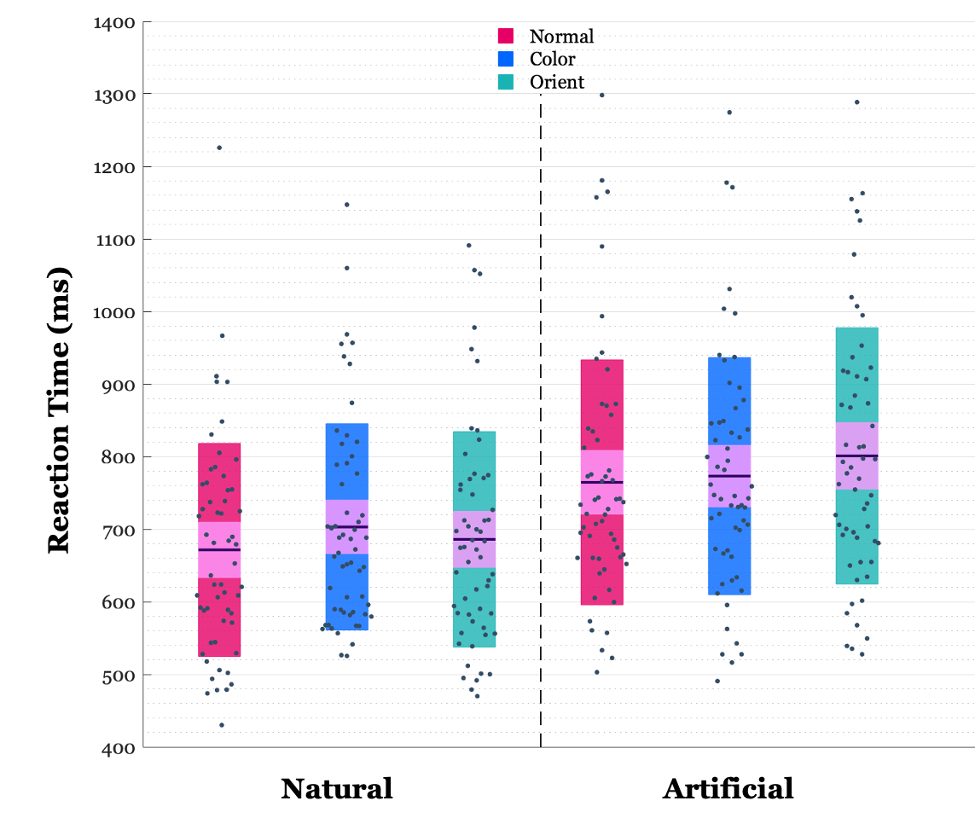
**

Fig. A.2. *Scatterplot of reaction time averaged across all trials per subject, plotted by category and condition.* Each dot represents the mean reaction time for an individual subject. The color of each box corresponds to condition (see legend), the expanse of the box represents one standard deviation, the pink middle strip represents the standard error of the mean (SEM) for the 95% confidence interval, and the purple line represents the mean.
