# Supplementary material for: No fruits without color: Cross-modal priming and EEG reveal different roles for different features across semantic categories
Source: PLoS One. 2021 Apr 14;16(4):e0234219. doi: 10.1371/journal.pone.0234219 (PMC8046255; doi:10.1371/journal.pone.0234219)
Supplement: S8 File — (RTF) [file pone.0234219.s011.rtf]

EEG .mat files per condition and category, averaged across trials within each category/condition	-FILES contained: artcolor.mat, artnorm.mat, artorient.mat, natcolor.mat, natnorm.mat, 	natorient.mat:		-These are .mat files of dimension 128 x 358 x 32 representing (channel x time point x 		participantEEG .mat channel file (Chan.mat) contains channel informationBehavioral data (Lexical_data) is a .txt file that contains trial-level information of reaction time and accuracy organized by participant, with condition and category nested within each participant.We also provide a commented R script containing our behavioral analysis that can be run on the accompanying Lexical_data.txt file. 
